# Recruitable volume is comparable in acute respiratory distress syndrome and in healthy lungs

**DOI:** 10.1186/cc12062

**Published:** 2013-03-19

**Authors:** CA Stahl, K Moeller, D Steinmann, D Henzler, S Lundin, O Stenqvist

**Affiliations:** 1 University Medical Center Freiburg, Germany; 2 Biomedical Engineering, Villingen-Schwenningen, Germany; 3 Klinikum Herford, Germany; 4 Sahlgrenska University Hospital, Gothenburg, Sweden

## Introduction

The application of PEEP is commonly used in acute respiratory distress syndrome (ARDS) and has been shown to improve oxygenation. To identify patients that most benefit from the application of PEEP, the discrimination of recruiters and nonrecruiters has been postulated by Gattinoni and colleagues [1]. Recently, Dellamonica and colleagues [2] presented a method to predict alveolar recruitment. We hypothesised that the amount of recruitable volume allows the discrimination between ARDS patients and patients with healthy lungs (HL).

## Methods

We recalculated the recruited volume (RV) in 25 patients with ARDS [3] according to the method proposed by Dellamonica and colleagues during an incremental PEEP manoeuvre (PEEP increased until the plateau pressure reached 45 cmH_2_O). RV was calculated as the change in end-expiratory lung volume minus total respiratory system compliance times the PEEP change (RV = ΔEELV - CTOT×ΔPEEP). For comparison, 12 patients with HL undergoing elective surgery in general anaesthesia were measured using the same protocol.

## Results

**Figure 1:**
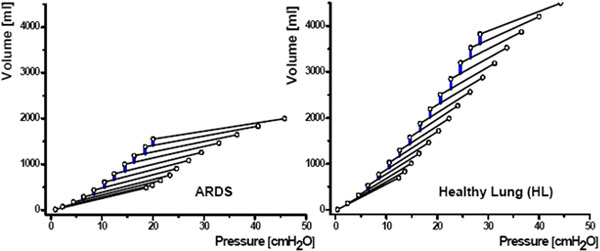


**Figure 2:**
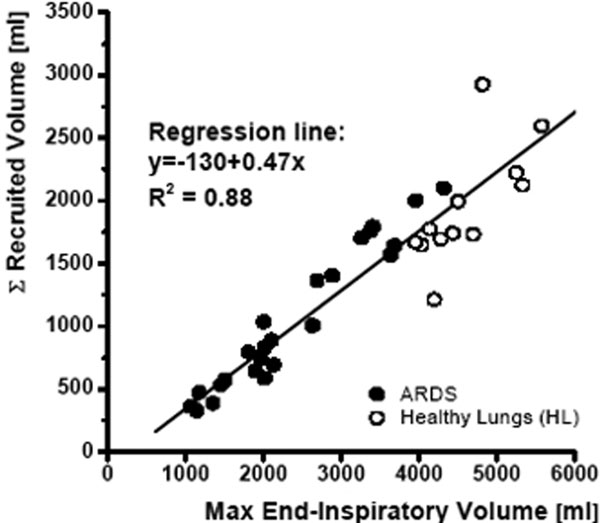


Both ARDS and HL patients exhibited typical P-V curves and stepwise recruitment (Figure 1). By raising PEEP from 0 to 12 cmH_2_O, ARDS patients recruited 331 ± 195 ml (mean ± SD) and HL patients 435 ± 43 ml. There was a strong correlation (*R*^2 ^= 0.88) of the total RV with the end-inspiratory volume at a plateau pressure of 45 cmH_2_O in both groups; that is, recruitment was found to the same extent in both groups (Figure 2).

## Conclusion

The relative contribution of RV to lung volume gain is similar in ARDS and in patients with healthy lungs. Our results question the relevance of recruitability as defined by Dellamonica and colleagues as a typical phenomenon of ARDS, but support the baby lung concept, as the recruited volume was closely related to the size of the lung.
